# Supplementary material for: Targetable ERBB2 mutation status is an independent marker of adverse prognosis in estrogen receptor positive, ERBB2 non-amplified primary lobular breast carcinoma: a retrospective in silico analysis of public datasets
Source: Breast Cancer Res. 2020 Aug 11;22:85. doi: 10.1186/s13058-020-01324-4 (PMC7422515; doi:10.1186/s13058-020-01324-4)
Supplement: Supplementary file 1 — Additional file 1: Supplemental Figure S1. (A) 10-year OS KM plot stratified by individual study cohort: blue – METABRIC, solid gray – TCGA, dotted gray – MSK-IMPACT; (B) 10-year OS KM plot stratified by study category: blue – long follow-up (METABRIC), gray – short follow-up (TCGA and MSK-IMPACT combined); Logrank p = 0.225. [file 13058_2020_1324_MOESM1_ESM.pdf]

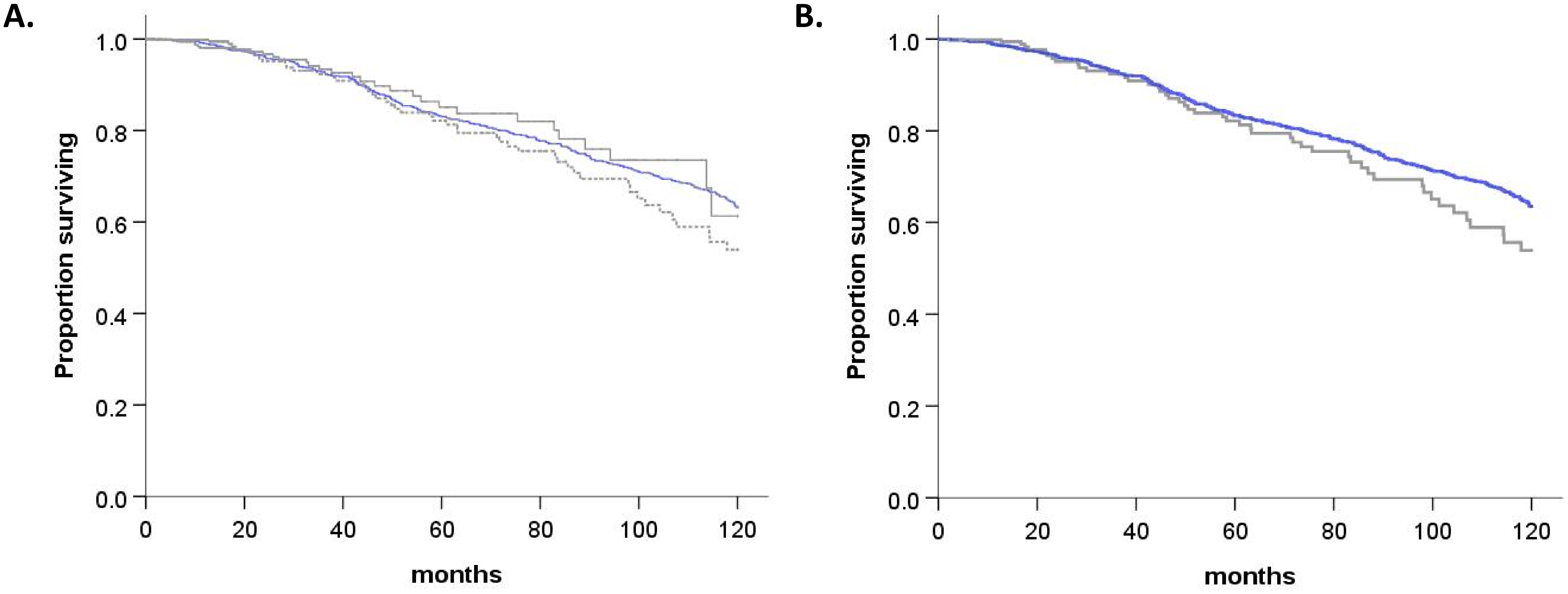

Figure S1: (A) 10-year OS KM plot stratified by individual study cohort: blue – METABRIC, solid gray – TCGA, dotted gray – MSK-IMPACT; (B) 10-year OS KM plot stratified by study category: blue – long follow-up (METABRIC), gray – short follow-up (TCGA and MSK-IMPACT combined); Logrank p=0.225
