# Supplementary material for: Targetable ERBB2 mutation status is an independent marker of adverse prognosis in estrogen receptor positive, ERBB2 non-amplified primary lobular breast carcinoma: a retrospective in silico analysis of public datasets
Source: Breast Cancer Res. 2020 Aug 11;22:85. doi: 10.1186/s13058-020-01324-4 (PMC7422515; doi:10.1186/s13058-020-01324-4)
Supplement: Supplementary file 2 — Additional file 2: Supplemental Tables. (S1) list of genes in the novel (N = 20) and established (Desmedt et al [31], N = 24) gene signatures of HER2 activity; (S2 and S3) top 500 DEGs by WAD score stratified into genes that are up-regulated (S2) and down-regulated (S3) for METABRIC ERBB2 amplified vs. non-amplified (ERBB2amp), METABRIC ERBB2mut vs. wild-type (ERBB2mut) and oncERBB2mut vs. wild-type (oncERBB2mut), and TCGA HER2+ vs. HER2- (TCGA HER2+). [file 13058_2020_1324_MOESM2_ESM.pdf]

Table S1: HER2 gene signatures: (i) Novel and (ii) Established by Desmedt *et al* 2008 (31). Pearson R [neratinib] is the correlation with neratinib response using a pharmacogenomic model of breast cancer cell lines (accessed online *via* CellMinerCDB). *indicates significantly correlated genes. n/a = data not available in CellMinerCDB

Genesig	*N*	Gene ID	Pearson R [neratinib]	*p*-value
(i) Novel	20	*ERBB2*	0.77	1.90E-08*
		SPDEF	0.45	4.20E-03*
		TFAP2B	0.2	0.24
		CD24	0.38	0.019*
		SERHL2	0.41	0.0097*
		CNTNAP2	0.12	0.47
		RPL19	0.29	0.073
		CAPN13	0.51	1.00E-03*
		RPL23	0.22	0.18
		LRRC26	n/a	n/a
		PRODH	0.42	9.00E-03*
		GPRC5C	0.44	0.0056*
		GGCT	0.38	1.90E-02*
		CLCA2	0.31	5.70E-02
		KDM5B	0.33	4.20E-02*
		SPP1	-0.25	1.30E-01
		PHLDA1	-0.54	5.30E-04*
		C15orf48	0.06	7.10E-01
		SUSD3	-0.09	5.90E-01
		SERPINA1	0.14	4.10E-01
(ii) Established	24	*ERBB2*	0.77	1.90E-08*
		PERLD1	0.77	1.20E-08*
		PSMD3	0.33	0.04*
		PNMT	0.33	4.20E-02*
		GSDML	0.4	1.40E-02*
		CASC3	0.26	0.11
		LASP1	0.32	0.049*
		WIPF2	0.27	9.70E-02
		EPN3	0.42	8.50E-03*
		PHB	0.38	0.019*
		CLCA2	0.31	5.70E-02
		ORMDL2	0.06	0.74
		RAP1GAP	0.53	0.00059*
		CUEDC1	0.09	0.61
		HOXC11	0.2	0.23
		CYP2J2	0.45	0.0044*
		HGD	0.14	0.39
		ABCA12	0.07	0.67
		ATP2C2	0.42	0.0096*
		ITGA3	0	0.98
		CEACAM5	0.4	0.012*
		TMEM16K	0.15	0.37
		NR1D1	n/a	n/a
		SNX7	-0.28	0.092
		FJX1	-0.26	0.12
		KCTD9	-0.11	0.53
		PCTK3	-0.04	0.83
		CREG1	0.17	0.3

Table S2: Up-regulated genes from the top 500 DEGs for each comparison by WAD score

**METABRIC *ERBB2*amp**	**METABRIC *ERBB2*mut**	**METABRIC onc*ERBB2*mut**	**TCGA HER2+**
ERBB2	*PIP*	*ANKRD30A*	*ERBB2*
GRB7	*CYP4Z1*	*CYP4Z1*	*SCGB2A2*
PGAP3	*PROM1*	*LRRC26*	*SPDEF*
GSDMB	*CD24*	*PPP1R1B*	*FOXA1*
PPP1R1B	*GSTM2*	*PIP*	*TFF3*
CD24	*CYP4X1*	*LIMCH1*	*TFAP2B*
LAD1	*ANKRD30A*	*UGT2B7*	*ABCC11*
ORMDL3	*PPP1R1B*	*COL4A5*	*ALDH3B2*
STARD3	*BAMBI*	*UGT2B11*	*FGFR4*
S100P	*AGL*	*BAMBI*	*BCAS1*
C17orf37	*IRX2*	*CFB*	*CEACAM6*
LRRC26	*VTCN1*	*TFAP2B*	*KCNK1*
S100A8	*LIMCH1*	*CYP4X1*	*CEACAM5*
MED1	*NDP*	*RAP1GAP*	*ORMDL3*
CBX2	*LAD1*	*C9orf152*	*C17orf37*
CALML5	*RPL23*	*ABCC11*	*CD24*
MED24	*C9orf152*	*AGR3*	*PGAP3*
KRT7	*TFAP2B*	*AGL*	*GRB7*
RPL19	*DSP*	*DDX17*	*HOXC10*
SDC1	*KDM5B*	*GSTM2*	*AGR2*
C1orf106	*PRODH*	*DACH1*	*C9orf152*
DQ893812	*RPL9*	*NFIA*	*AR*
IDH2	*RPL39*	*CAPN13*	*STARD3*
FBXL20	*CLCA2*	*FOXA1*	*PRR15*
BAMBI	*RPS27A*	*SLC12A2*	*SERHL2*
KCNK1	*LRRC26*	*SERHL2*	*PSMD3*
SUSD2	*RPS23*	*CLCA2*	*CNTNAP2*
TMEM132A	*NFIB*	*IRX2*	*GALNT6*
PSMD3	*RPS27*	*UGT2B28*	*TOB1*
PSMB3	*HMGCS2*	*DKFZp761K0816*	*DHRS2*
MLLT6	*XIST*	*PRODH*	*ITGB6*
CDC20	*U79293*	*SCGB1D2*	*SCGB1D2*
SOX11	*UAP1*	*KLHDC2*	*HS6ST3*
GLYATL2	*NQO1*	*HMGCS2*	*KITLG*
SLC7A5	*RPS4X*	*HPX*	*PRLR*

TFRC	*RAB27B*	*SH3BGRL*	*ACSS3*
RPL23	*RPL37A*	*LCOR*	*RPL19*
DBNDD1	*DUSP6*	*NOSTRIN*	*ABCA12*
S100A9	*DDX17*	*MUC1*	*MED1*
PROM1	*ARGLU1*	*RTN4*	*HOXC11*
UBE2C	*KRT7*	*SPINK8*	*TIMP3*
G6PD	*GSTP1*	*ARGLU1*	*CMBL*
LASP1	*UGDH*	*PPFIBP2*	*C1orf115*
SCD	*MAP3K1*	*HNRNPA1*	*ETNK2*
CYB561	*MPZL2*	*EFCAB4A*	*MUM1L1*
CDK12	*SLC38A2*	*MAP3K1*	*CYP2J2*
SERHL2	*AGR3*	*SLC38A1*	*FASN*
ULK1	*LCOR*	*SMARCA2*	*COL12A1*
PHGDH	*RPL5*	*TFF3*	*TOM1L1*
NOTCH3	*PROM2*	*PJA2*	*RNF43*
CAPN13	*ARL6IP5*	*GPC4*	*CYP4Z2P*
PFKP	*DCP2*	*TBC1D9*	*HOXB13*
CAPS	*ZFP36L1*	*UBE3A*	*HOTAIR*
KRT8	*EFCAB4A*	*ATP1B1*	*CLTC*
CDR2L	*OGT*	*GPRC5C*	*IGFBP5*
TUBB3	*SPINK8*	*REEP1*	*NCAM2*
TLCD1	*RPL31*	*ACSL3*	*TC2N*
DBN1	*CCDC14*	*TSPAN13*	*DUSP4*
CLCA2	*F11R*	*THUMPD1*	*CAPN13*
N4BP2L2	*BNIPL*	*UAP1*	*C1orf64*
FOXO3	*GSTM1*	*MOBKL3*	*GPR160*
FLJ40504	*RPL7*	*RAB27B*	*CDK12*
PSME4	*PTMA*	*CXADR*	*POF1B*
CASC3	*SHISA2*	*LMTK3*	*SCGB2A1*
TOP2A	*MOBKL3*	*ARL6IP5*	*SLC4A8*
C11orf80	*RAP1GAP*	*RPL9*	*MOSC1*
TFAP2B	*UGT2B11*	*KIAA1370*	*MMP11*
EIF2C2	*GMFB*	*MLPH*	*HPGD*
PDXK	*YES1*	*LANCL1*	*TSKU*
HMGB3	*FLJ40504*	*PROM2*	*TRIM36*
TRIB3	*PPFIBP2*	*RPS23*	*MGST1*
ADAM15	*CNN3*	*INPP5J*	*AIM1*
S100A16	*RPL19*	*PROM1*	*THRSP*

ASS1	*GOLGA8B*	*U79293*	*SIX1*
ASPM	*RPL26*	*NTN4*	*GSTM3*
GPRC5C	*ZNF827*	*REEP6*	*MFAP5*
PPP1R10	*PHGDH*	*GOLGA8B*	*ZNF652*
CACNB3	*PRKAR1A*	*SLC38A2*	*RPL23*
HES4	*STYX*	*ECHDC2*	*LRRC26*
PPP1R14B	*LOC442454*	*TC2N*	*ERBB4*
RANGAP1	*A2M*	*HIGD1A*	*COL11A1*
FLOT2	*RPS7*	*DUSP6*	*POSTN*
PROM2	*NACA*	*KIAA1671*	*MOSC2*
GALE	*SERHL2*	*AZGP1*	*NPNT*
YES1	*ATP1B1*	*TP53INP1*	*PRODH*
UBQLN4	*MGEA5*	*GMFB*	*PECI*
PNMT	*COL4A5*	*ALCAM*	*RIMS1*
FAT1	*RPS3A*	*REEP5*	*INPP4B*
CDC45	*PABPC1*	*CRIP2*	*SLC9A2*
KDM5B	*CAMSAP1L1*	*ACACA*	*CXCL17*
EZR	*P4HA1*	*DSTN*	*RND1*
NME1	*TROVE2*	*OGT*	*CREB3L4*
LFNG	*REEP5*	*ZNF827*	*SLC2A10*
SMARCA4	*CXADR*	*EIF3A*	*PLCB4*
PRODH	*EIF3A*	*ZNF91*	*FLT3*
PRSS8	*RTN4*	*ENPP5*	*MGAT4A*
GGCT	*LUC7L3*	*RPL23*	*CTNND2*
PCGF2	*ZRANB2*	*PPA2*	*RET*
NUSAP1	*SEC61G*	*MYB*	*AFF3*
RPS6KB2	*EZR*	*SCGB2A2*	*GATA2*
C10orf81	*UBE3A*	*KCNMA1*	*SDR16C5*
RPS6KB1	*GGCT*	*ESRRG*	*C17orf28*
SLC9A3R1	*UGT2B7*	*N4BP2L2*	*MYO1B*
CR613620	*GSTO2*	*PDE4B*	*FA2H*
ITPR3	*LANCL1*	*MGEA5*	*CDC6*
SLC38A2	*MGP*	*HNRNPA0*	*IYD*
LUC7L3	*HMGN2*	*CADPS2*	*MSL1*
MIF	*XPR1*	*CYP4Z2P*	*TMEM150C*
SLC25A1	*TRA2B*	*CKMT1B*	*SEPP1*
GAPDH	*SH3BGRL*	*ABLIM1*	*ALCAM*
INF2	*KLHDC2*	*ZNF302*	*NAP1L2*

KRT81	*RFWD2*	*LGALS8*	*PVALB*
TRAF3IP2	*SERTAD4*	*TTC3*	*ERGIC1*
PPFIBP2	*PPA2*	*SH3BP4*	*XBP1*
LOC388152	*MIF*	*FAM98A*	*GPRC5C*
PHB	*ZNF302*	*MYO1B*	*HPX*
TMC6	*ACLY*	*FH*	*DACH1*
SNF8	*ANO1*	*KDM5B*	*PHLDB2*
AKT1	*LARP4*	*ECI2*	*FAM3B*
38777	*GPC4*	*XPR1*	*SLC16A14*
C17orf28	*CDC42SE1*	*UGDH*	*SERHL*
KIAA0182	*INPP5J*	*ZFP36L1*	*ATP13A4*
INTS8	*SREK1*	*F11R*	*KRT18*
SBK1	*CALM2*	*PNPLA7*	*NQO1*
CCDC6	*ERBB2*	*SREK1*	*FBN1*
VMP1	*THUMPD1*	*AK025793*	*MAP9*
TP53I13	*HSPA2*	*FEM1C*	*OSBPL6*
HMGCS1	*UBQLN4*	*FAM13B*	*SDC1*
PTTG1	*RBM25*	*KTN1*	*TM7SF2*
FAM38A	*SCGB1D2*	*LRIG1*	*PRKACB*
MUC1	*PPP1CB*	*DBNDD1*	*RHOB*
SNAR-A3	*TP53INP1*	*DBI*	*TMEM86A*
STIP1	*SMARCA2*	*CMPK1*	*LUM*
C22orf36	*PJA2*	*GLCE*	*FAM84B*
MMP9	*ROD1*	*CR610863*	*SLC7A2*
CMTM4	*SNX14*	*ARHGAP32*	*LASS6*
PTRH2	*STK36*	*PRKAA1*	*SBNO1*
LIMCH1	*NKTR*	*SLC26A3*	*CRISP3*
NFIB	*RPS6*	*XIST*	*PHB*
LDLR	*MYO1B*	*DCP2*	*UNC5A*
LMTK3	*SCARB2*	*NXPH1*	*F13A1*
FAM110A	*CYB5R2*	*IGSF21*	*PNMT*
PXDN	*GDI2*	*RPS27A*	*S1PR3*
COL7A1	*FH*	*RPS4X*	*INPP5J*
CARM1	*SLC30A7*	*TM9SF2*	*ALOX15B*
HMGA1	*MGC24103*	*PRRC1*	*CD55*
RAP1GAP	*FAM98A*	*ATP1A1*	*EPN3*
EFCAB4A	*RNF144B*	*CUL4A*	*SCD*
CWC25	*SLC38A1*	*TRA2B*	*CREB3L1*

FER1L4	*HNRNPA0*	*FKBP3*	*GGCT*
RAPGEFL1	*KTN1*	*CR613620*	*CLCA2*
CCNB2	*ETS1*	*ZRANB2*	*RPS6KB1*
SEMA4B	*TC2N*	*TMEM62*	*TMC5*
MRPL27	*ACACA*	*C3orf14*	*PAX9*
NCK2	*PRRC2C*	*LUC7L3*	*VCAN*
CNTNAP2	*NARS*	*CCNO*	*SPTLC2*
EPPK1	*KIAA0528*	*MKNK2*	*CXCL9*
C4orf48	*RHOB*	*ARPP19*	*ACSL3*
RARA	*DCUN1D4*	*PTEN*	*CBX2*
PABPC1	*MAP3K6*	*ANAPC13*	*PDE10A*
EDARADD	*MYB*	*NARS*	*IQGAP2*
SLC5A6	*TMEM62*	*KIAA1598*	*GATA3*
SPDEF	*INTS8*	*CCDC6*	*SLC16A2*
LPCAT1	*RPS15*	*A2M*	*CDH2*
MLLT4	*LEMD3*	*EVL*	*WIPF2*
YWHAG	*PTPRK*	*SPDEF*	*MORC4*
NPIPL3	*LGALS8*	*SNX14*	*PRR15L*
PSMC5	*EIF4A2*	*PTMA*	*MLPH*
TRPV6	*UBE2Q2*	*CAMSAP1L1*	*KIAA1244*
PODXL2	*TRIP12*	*C7orf41*	*EMB*
DDIT4	*PPP1R3C*	*NPTN*	*COL10A1*
FLAD1	*YWHAG*	*RPL3*	*KIAA2022*
ABLIM1	*THOC2*	*RPL5*	*ENPP1*
LCN2	*EIF4B*	*CBX6*	*SOX11*
CDCA5	*SOCS2*	*RPS26*	*NRK*
FAM60A	*NOTCH3*	*DCTN4*	*F2RL2*
ARFGAP1	*STK32B*	*TJP1*	*COL6A3*
RCC2	*C8orf59*	*RCOR3*	*ADRA2C*
SNAPC4	*MST4*	*TSC1*	*PARM1*
VPS37B	*CR613620*	*CALM2*	*C2orf72*
COMT	*RPL3*	*KIAA0353*	*NME1*
SPINK8	*RPS11*	*ERBB3*	*SCARB2*
S100A11	*KCNMA1*	*VANGL2*	*F7*
E2F2	*TTC3*	*FLJ40504*	*FBXL20*
RHPN2	*UGT2B28*	*SAR1A*	*TMEM45B*
MTMR4	*FNBP1L*	*RHOB*	*RHOBTB3*
CHFR	*SRSF1*	*NACA*	*GGT1*

FAM98A	*CYP4Z2P*	*FTH1*	*FAM108C1*
	HNRNPA1	*ESR1*	*SRD5A3*
	NOSTRIN		*PCDHB8*
	FEM1C		*AGL*
	TMEM132A		*TGFBR1*
	FBXO11		*MED24*
	PRKAA1		*KIAA1324*
	SOX4		*ARHGAP8*
	RHPN2		*CLGN*
	CNTNAP2		*ST3GAL5*
	RCOR3		*HS6ST2*
	PERP		*IDI1*
	CDC16		*SPRED2*
	HNRPDL		*GSR*
	ABCC11		*DHX40*
	FOXA1		*KIAA1199*
	SUZ12		*TMTC3*
	ACSL3		*GPNMB*
	C1orf106		*ISOC1*
	RPL15		*THSD4*
	WASL		*G3BP2*
	PSME4		*ACSL1*
	TFAP2A		*CATSPERB*
	EXOSC10		*MARCKS*
	LPP		*TPD52L1*
	STX6		*GPR137B*
	SERBP1		*CD164*
	SUCLG2		*PDE4B*
	CCBL2		*FN1*
	PRRC1		*PP14571*
	DQ893812		*ACER3*
	DARS		*TSC22D3*
	RPL36A		*ATP2C2*
	ZBTB33		*MEGF9*
	ARHGEF2		*TMEM49*
	TEAD2		*CCDC125*
	ZZZ3		*KDM5B*
	SPRED1		*ANKRD50*

CMPK1	*PCDH20*
KIAA0353	*MUCL1*
UHMK1	*SYNPO2*
DSTN	*APPBP2*
FAM13B	*GPRIN3*
ECI2	*EPS8*
COX7B	*LRRC59*
NLRP2	*ABCC4*
SLC26A3	*CPE*
SLC12A2	*ASPN*
GTF2H5	*REEP1*
DBI	*SORD*
SMARCC1	*BMP7*
CUL4A	*HEATR6*
CRABP2	*XIST*
LRRC1	*ANKRD22*
AK130741	*GRIP1*
DHX15	*NFIA*
EFNB2	*PLEKHA6*
ENPP5	*MSMB*
MTPN	*TGFB3*
CCDC6	*CACNA1H*
DDX3X	*TMEM63C*
TM9SF3	*USP32*
CADPS2	*ODZ2*
C6orf115	
RHOA	
PTEN	
SH3BP4	
TJP1	

Table S3: Down-regulated genes from the top 500 DEGs for each comparison by WAD score

**METABRIC**	**METABRIC**	**METABRIC**	**TCGA**
**ERBB2amp**	**ERBB2mut**	**oncERBB2mut**	**HER2+**
S100A13	*KCTD12*	*PSMC1*	*SGK223*
ATP1B1	*HIST2H4B*	*HERPUD1*	*HIST1H2BJ*
PMP22	*ATF3*	*KIAA1949*	*PTPN20B*
CTNNB1	*ATG4B*	*IRF1*	*ABLIM2*
PDZK1	*UBC*	*TPM3*	*SLC6A9*
DNAJB6	*TCEAL1*	*LIPA*	*SELM*
EEF1A1	*TXNDC5*	*VAMP5*	*C8orf46*
SNHG5	*PLAU*	*GIMAP7*	*SDC2*
FSIP1	*IFI30*	*GNLY*	*CPXM2*
ISCU	*DCTN3*	*MAF*	*FADS3*
IFITM3	*GYPC*	*IGFLR1*	*MAPK12*
NFE2L2	*EIF4A1*	*C10orf116*	*ACP5*
MBOAT1	*SMPDL3A*	*FTL*	*ZNF439*
MTCP1NB	*FAIM3*	*CRTAP*	*SYN1*
IAH1	*IGSF6*	*FKBP11*	*DIRAS1*
ALDH6A1	*LAPTM5*	*C9orf46*	*GSTP1*
HSPB11	*HBEGF*	*MGC87042*	*PDE4A*
GNG11	*DISC1*	*FSTL1*	*TRIM9*
PSMC1	*FKBP14*	*CPXM2*	*A4GALT*
PDGFRL	*IRF1*	*RBP7*	*TSPYL5*
TCTN3	*HLA-E*	*LYN*	*NELL2*
GZMK	*PGAM1*	*EPDR1*	*RTP4*
HNRNPR	*TPM3*	*ARF4*	*STAC*
CCL15	*CPXM2*	*RRAS*	*PANX2*
LOC401397	*CX3CR1*	*PODN*	*ARHGEF19*
RNF13	*RARRES3*	*C1orf151*	*PTHLH*
ARF4	*CD86*	*NEDD8*	*SLPI*
GPR34	*IRX3*	*PLAT*	*CAND2*
CTSK	*TMED9*	*GZMA*	*RTN4RL1*
HSD17B11	*KDELR2*	*FOXC1*	*LTBP3*
FGD3	*ACOT2*	*QPCT*	*APBB1*
PRNP	*IL10RB*	*RIPK2*	*SETBP1*
GPNMB	*ADAP2*	*PFKP*	*RBM24*
COMMD10	*ALDH3A2*	*KDELR2*	*ADCY9*
SLC7A2	*SLC2A3*	*LGALS1*	*KCTD14*

OAT	*CST1*	*SIK1*	*VANGL2*
IGFBP6	*CTGF*	*CYP4B1*	*CALML5*
RNU11	*UGCG*	*MS4A7*	*GPT*
SELM	*EVI2B*	*LY6E*	*REEP2*
CFH	*PRIC285*	*RPL8*	*ACOT11*
TMEM14A	*ITM2B*	*FILIP1L*	*HDAC5*
SESTD1	*SRSF10*	*SERPINH1*	*ARHGEF10L*
CSAD	*TMEM140*	*DPT*	*CD44*
RPL21	*FST*	*IGFBP7*	*KLHL21*
ASH2L	*RAB5C*	*CD248*	*ATHL1*
HSD17B4	*TTC39C*	*AKR1B1*	*FBXL16*
JUNB	*ECHDC3*	*PAPD5*	*C4orf7*
RASD1	*TIMP3*	*SGCE*	*KCNN4*
BNIP3L	*LDOC1*	*DRAM1*	*NRBP2*
PGCP	*LST1*	*GPX1*	*TACSTD2*
TMED10	*RAD51C*	*KRT19*	*CDSN*
CIDEC	*PPIC*	*C3*	*PAM*
ARPC3	*SELS*	*IFI30*	*PGR*
C1S	*C1orf162*	*ALDH1A1*	*MMP9*
ANKRD30A	*GRB2*	*ITM2C*	*TRIM47*
NFU1	*IGFBP4*	*SLC7A2*	*CDHR3*
SDCBP	*SLC7A5*	*CEBPB*	*UBXN11*
CCL3	*TMEM14A*	*CLIC6*	*BCL11A*
RBBP4	*S100A4*	*S100P*	*COL9A3*
SIAH2	*DOCK2*	*GGH*	*PLOD3*
NDUFS4	*MAP1LC3A*	*CD2*	*CSF3R*
ARMC10	*GADD45B*	*ANXA2*	*AGTRAP*
TCEA3	*SPATS2L*	*DOCK2*	*TCIRG1*
ELOVL5	*PIGT*	*COL3A1*	*FLNB*
SEC23B	*SLC15A3*	*EVI2B*	*LGR6*
LITAF	*CISD1*	*PRDX4*	*PRRT2*
ARL1	*CXCL12*	*CFH*	*SCARA3*
TNFAIP6	*UBD*	*ALDH3A2*	*NDRG2*
C6orf48	*CREG1*	*TIMP1*	*TAT*
NSA2	*TBXAS1*	*BHLHE40*	*PLEKHG4*
PPIC	*CRTAP*	*ID3*	*STEAP3*
CD52	*PDGFRL*	*CD79A*	*PLCH2*
HERPUD1	*PLEK*	*FST*	*TNFRSF14*

FCGR2A	*TRIB1*	*ANGPTL4*	*ERRFI1*
C13orf15	*FNDC1*	*TMED4*	*KRT14*
ACYP2	*PSMC4*	*FNDC1*	*MAPK15*
ITM2A	*TFF1*	*NCRNA00152*	*CCDC74B*
RPL26	*CXCR7*	*CTSB*	*DUOX1*
FCGBP	*FPR1*	*CD68*	*HIST2H4A*
EGR2	*TMEM119*	*THOC4*	*IL22RA1*
SRPX	*LY6E*	*CD86*	*SDK2*
PHLDA1	*LYN*	*RAC2*	*GSDMD*
PODN	*CSF2RA*	*IGLL3P*	*C10orf10*
METTL5	*FKBP1A*	*IL32*	*SPP1*
SPP1	*SLC7A7*	*CAV2*	*PI15*
GYPC	*PSMB8*	*CXCL16*	*KLK11*
TPM1	*NGRN*	*NNMT*	*KRT6B*
TCEA1	*HERPUD1*	*MMP11*	*KRT15*
SRSF10	*AIF1*	*HLA-DOA*	*CD164L2*
GSTM2	*ARPC3*	*PPIC*	*COL22A1*
UBE2L6	*RET*	*KLF6*	*CD320*
TIMP3	*AGTR1*	*NKG7*	*SHC4*
NR4A2	*ARF4*	*RAD51C*	*PHLDA1*
TSC22D1	*RAB32*	*C13orf15*	*ESPN*
MGC87042	*PLOD2*	*BTG3*	*FOXC1*
PLBD1	*PSMC1*	*COL6A1*	*CDON*
C4B	*CRISPLD2*	*GEM*	*SAA1*
ADH1A	*QPCT*	*FPR1*	*RGMA*
CCL2	*PTPN1*	*PQLC3*	*CITED1*
DQ896551	*PPAPDC1B*	*HLA-DMB*	*PPP4R4*
PLIN4	*C3*	*TCRVB*	*DSC3*
ZFAND6	*GCLM*	*FERMT3*	*PLOD1*
THBS1	*TCRA*	*SPCS2*	*VTCN1*
RUNDC1	*TSPAN3*	*SUSD3*	*FOXI1*
LHFP	*ACP5*	*ACO1*	*CA2*
C10orf32	*NINJ2*	*PXMP2*	*ECE1*
FUCA1	*PLA2G16*	*IGFBP4*	*IRAK1*
VBP1	*RGS16*	*CD3D*	*CRABP1*
ATP6V0E1	*CSF1R*	*GBP1*	*CX3CL1*
BEX2	*C2CD4B*	*SERPINE1*	*CRIM1*
AKR1B1	*FOLR2*	*DQ896551*	*SOSTDC1*

SYF2	*CYBA*	*GAS1*	*SERPINA6*
IGFBP7	*LIPA*	*CREG1*	*HRASLS5*
SUB1	*WISP2*	*CD83*	*SGK3*
CYB5A	*STOM*	*PHYH*	*LYPD5*
NELL2	*CMTM8*	*HLA-DPB1*	*PTPRU*
C3orf10	*HLA-DOA*	*CALB2*	*PGCP*
ESD	*GLRB*	*ECHDC3*	*PDZK1IP1*
OCIAD1	*C11orf96*	*UBE2L6*	*RCOR2*
PDK4	*COL12A1*	*PEMT*	*KLK10*
JUN	*GLIPR1*	*ATP6V1B1*	*MYH14*
TMEM59	*IFIT1*	*CSF1R*	*PROM1*
TSG101	*SPP1*	*FAP*	*KAZ*
RSL1D1	*CTSL1*	*RNASE6*	*BAI2*
TMEM126A	*SNAR-A3*	*MDK*	*NEK10*
ZG16B	*IGLL1*	*RDH10*	*TMEM139*
CNIH	*SPCS2*	*SLC7A7*	*FUT2*
FAM129A	*CTSB*	*IL6*	*IL27RA*
KLHL5	*GNG10*	*JUN*	*PYGL*
NGFRAP1	*HLA-DMA*	*RGS16*	*CBLC*
AGR2	*C1orf151*	*SLC16A3*	*RAMP3*
LAMP2	*SERINC3*	*GADD45B*	*HIST2H2BE*
RGS2	*SMOC2*	*C1orf162*	*GNA15*
GEM	*KDELR3*	*CMTM7*	*MAOA*
CD151	*GRIA2*	*TMEM119*	*MESP1*
PSMG2	*HCST*	*EPSTI1*	*SLC34A2*
F13A1	*HCLS1*	*COL5A2*	*MIA*
ANXA1	*MZB1*	*GYPC*	*MGP*
CD14	*MMP9*	*TGFBI*	*SYT8*
ALG13	*COL11A1*	*EIF4A1*	*NFASC*
MT1E	*FAM174A*	*S100A16*	*RLTPR*
RNASE6	*BHLHE40*	*CAV1*	*NFIX*
ACADM	*RNU11*	*LAPTM5*	*AGRN*
SH3BGRL	*RASD1*	*PLEK*	*CBS*
NFIX	*EIF4H*	*SERPING1*	*S100A6*
MS4A7	*FAM134B*	*EGR2*	*PPANxyxP2RY11*
PLA2G16	*EPSTI1*	*SLC15A3*	*STC2*
ITPR1	*SLC7A2*	*NINJ2*	*RTN1*
SEZ6L2	*RNASE6*	*CTSG*	*MALL*

TGFBR3	*COL8A1*	*HCLS1*	*EFNA5*
SERINC3	*FCGRT*	*TXNDC5*	*ZNF385B*
AIF1	*C1QA*	*GNG10*	*PKP1*
VPS25	*TAP1*	*FAM164A*	*GRIA2*
FCGRT	*HM13*	*NDRG1*	*CKB*
CUTA	*CPA3*	*PITX1*	*PLCE1*
WNK4	*STC1*	*TAP1*	*LAMB3*
UGCG	*CTSK*	*GPX3*	*NYNRIN*
HNMT	*BRI3*	*THRSP*	*C1orf130*
PLEKHB2	*SFRP2*	*ANXA1*	*AHNAK2*
CCL3L3	*HIST1H2AC*	*SMOC2*	*FAM46B*
GLRB	*CD68*	*PLBD1*	*NBPF4*
NPEPPS	*HLA-DPB1*	*FAIM3*	*HIST2H2AA3*
DCTN6	*SYT13*	*GPNMB*	*ZSCAN18*
GJA1	*CD83*	*EGR1*	*BST2*
HNRNPA1L2	*LGMN*	*IER3*	*TF*
CD74	*AKR1B1*	*TAGLN*	*C15orf48*
DISC1	*CTSC*	*IFI6*	*SFRP1*
EIF4H	*PLAUR*	*NMB*	*CTSD*
TMED4	*APOC1*	*PIM2*	*TM4SF1*
MAOA	*TNFAIP6*	*SLCO2B1*	*ENO2*
PKIB	*JUNB*	*IGJ*	*CDK18*
STOM	*RAB31*	*BRI3*	*ADCY1*
FABP4	*CCL8*	*RNU11*	*CYP4F11*
CILP	*SLCO2B1*	*GRN*	*CHI3L1*
TSPAN3	*PHYH*	*FPR3*	*TTC22*
C11orf1	*LAPTM4B*	*RASD1*	*TUBB3*
PAIP2	*PTGES*	*CCL3*	*IL34*
RPL22	*CILP*	*AIF1*	*ZNF238*
PIGY	*DCN*	*ISG15*	*ANGPTL4*
PXMP2	*TMED4*	*LGALS3BP*	*SUSD3*
FCER1A	*UBE2L6*	*PDGFRL*	*DMKN*
SMOC2	*HIST2H2AC*	*PTGES*	*CAPG*
PROSC	*TNFSF13B*	*MXRA5*	*PGBD5*
ITM2B	*PLAT*	*S100A8*	*TUBA4A*
COLEC12	*CCL3*	*CTSC*	*LPPR3*
RAB5C	*ZFP36*	*MRPS30*	*MAPK8IP2*
CFB	*PKIB*	*C11orf96*	*C19orf33*

CHMP5	*CFD*	*GZMK*	*LY6E*
SGCE	*MFAP5*	*S100A6*	*MFSD6L*
CCL4	*HIST2H2AA4*	*MT1E*	*APOBEC3B*
HLA-A	*GPR183*	*RAB32*	*FXYD5*
SYBU	*FGL2*	*SLC2A3*	*CELSR2*
BMPR1B	*HLA-B*	*EMP3*	*TMEM101*
GNG10	*CXCL9*	*C2CD4B*	*SCNN1G*
AFF3	*C1R*	*SOD2*	*FAM198A*
CRYAB	*FCGR2A*	*CXCR7*	*ELF5*
SPCS2	*PLEKHB2*	*TRIB1*	*DBNDD2*
C1QB	*GRN*	*GLIPR1*	*FGD3*
ALDH1A1	*MYC*	*PHLDA1*	*WNK2*
CHPT1	*CD163*	*SAA1*	*PMAIP1*
SRP9	*CEBPD*	*TIMP3*	*GPRIN2*
IFI6	*HLA-DRA*	*FOLR2*	*C10orf81*
RBP7	*CXCR4*	*COL8A1*	*CA11*
TMEM119	*ITGB2*	*CILP*	*NDRG1*
LIPA	*PSMB9*	*COL12A1*	*ARTN*
IGFBP4	*MRPS30*	*C1S*	*C1orf173*
C10orf32-AS3MT	*CPVL*	*HLA-B*	*FAM20C*
LOC100506144	*CCL3L3*	*PLAUR*	*PLEKHB1*
CTSG	*EFEMP1*	*HLA-DMA*	*SOX10*
PPAP2A	*LGALS3BP*	*COMP*	*HR*
DNALI1	*CCL4*	*F13A1*	*SPEG*
PLAC9	*ISG15*	*LGMN*	*GABRP*
HLA-DPA1	*FCER1G*	*C1QA*	*COL2A1*
RNASE1	*FCGR1B*	*PLTP*	*LYNX1*
IGJ	*C15orf48*	*PLAU*	*KCNK5*
TMEM14C	*SFRP4*	*FCGR2A*	*AZGP1*
ASB13	*TPSAB1*	*FCGR1B*	*CRYAB*
MMADHC	*CXCL10*	*JUNB*	*EEF1A2*
CRTAP	*MS4A6A*	*HCST*	*NTN1*
FGL2	*CYR61*	*MZB1*	*GRB14*
C3	*CD14*	*SPP1*	*KRT16*
ATF3	*C1QC*	*C15orf48*	*SORBS2*
PIGT	*HLA-DPA1*	*PSMB9*	*TNNT1*
CTGF	*CD74*	*TNFSF13B*	*AGT*

CCNDBP1	*COL10A1*	*COL11A1*	*SECTM1*
GPR183	*IFI27*	*FABP5*	*MUC16*
FAM174A	*FOSB*	*HLA-DQB1*	*CITED4*
CEBPD	*BASP1*	*IFI27*	*ASPHD1*
ARMCX1	*TYROBP*	*HLA-DRB6*	*SFN*
SPCS1	*RNASE1*	*CTSL1*	*FBXO2*
CLSTN2	*SRGN*	*APOC1*	*CHI3L2*
CA2	*IFI6*	*CXCL12*	*CMTM7*
CAV1	*DKFZp686O16217*	*RARRES2*	*SCNN1B*
RPS27L	*C1QB*	*SERPINA1*	*FGFR2*
CPB1	*RGS1*	*CIDEC*	*S100A1*
TMEM101	*SERPINA1*	*FGL2*	*CCDC74A*
ZFP36	*HLA-A*	*CCL5*	*LAMC2*
HLA-DRA	*LYZ*	*FABP4*	*CFB*
TYROBP	*FOS*	*CRISPLD2*	*SERPINA1*
DACH1	*HLA-DQA1*	*CD74*	*MFI2*
TCEAL1	*DUSP1*	*CTGF*	*TFCP2L1*
CPVL	*abParts*	*RAB31*	*NPY1R*
CD163	*CDH1*	*ADM*	*FCGBP*
abParts	*HBA2*	*LUM*	*TMPRSS3*
BTF3	*HBB*	*SFRP2*	*SEZ6L2*
ALOX5AP		*WISP2*	*MUC5B*
C1R		*LAPTM4B*	*ANPEP*
FCER1G		*CCL3L3*	*WFDC2*
RBBP8		*MFAP5*	*SERPINA3*
CD36		*C1R*	*KRT23*
PRDX3		*CCL2*	
HLA-DQA1		*ITGB2*	
CREG1		*COL1A1*	
GATA3		*CD36*	
SEPP1		*FCGRT*	
BHLHE40		*CCL4*	
CX3CR1		*HLA-DRB1*	
C15orf48		*TNFAIP6*	
TBC1D9		*CEBPD*	
LUM		*CPA3*	
QDPR		*EFEMP1*	
MS4A6A		*HLA-DRA*	

MYC	*COL1A2*
SRGN	*GPR183*
HIST1H2AC	*CPVL*
CST3	*CYBA*
MGP	*ZFP36*
BCL2	*S100A4*
FAM134B	*MS4A6A*
CXCR4	*CTSK*
EFEMP1	*HLA-DPA1*
C9orf46	*CXCL9*
IL6ST	*DCN*
CITED4	*COL10A1*
GFRA1	*C1QC*
TPSAB1	*PLOD2*
CYBRD1	*PLIN2*
LYZ	*CXCR4*
CXCL12	*MMP9*
CFD	*PLIN4*
SPARCL1	*CD52*
SFRP2	*CFD*
ANG	*FCER1G*
RGS1	*ATF3*
THBS4	*CD163*
TFF1	*SRGN*
C1orf64	*UBD*
SUSD3	*MYC*
SCUBE2	*SFRP4*
SLC39A6	*CXCL10*
SERPINA1	*S100A9*
MRPS30	*TYROBP*
CPA3	*TPSAB1*
BTG2	*CCL8*
HBA2	*CD14*
SFRP4	*RNASE1*
CYR61	*C1QB*
DCN	*HLA-A*
MAPT	*RGS1*
CA12	*FOSB*

STC2	*CYR61*
RERG	*LYZ*
EGR1	*HBA2*
FOSB	*IGLL1*
NAT1	*HBB*
HBB	*DUSP1*
SERPINA3	*FOS*
DNAJC12	*HLA-DQA1*
DUSP1	*CDH1*
ESR1	*DKFZp686O16217*
FOS	*abParts*
AGR3	
