# Supplementary material for: Targetable ERBB2 mutation status is an independent marker of adverse prognosis in estrogen receptor positive, ERBB2 non-amplified primary lobular breast carcinoma: a retrospective in silico analysis of public datasets
Source: Breast Cancer Res. 2020 Aug 11;22:85. doi: 10.1186/s13058-020-01324-4 (PMC7422515; doi:10.1186/s13058-020-01324-4)
Supplement: Supplementary file 3 — Additional file 3: Supplemental Figure S2. Correlation between the observed response of breast cancer cell lines (N = 36) to neratinib and the response predicted by expression of (A) novel (N = 20) and (B) established (Desmedt et al [31], N = 24) gene panels. Plots were generated using CellMinerCDB online portal and cell line data from the BROAD Institute [34, 35]. [file 13058_2020_1324_MOESM3_ESM.pdf]

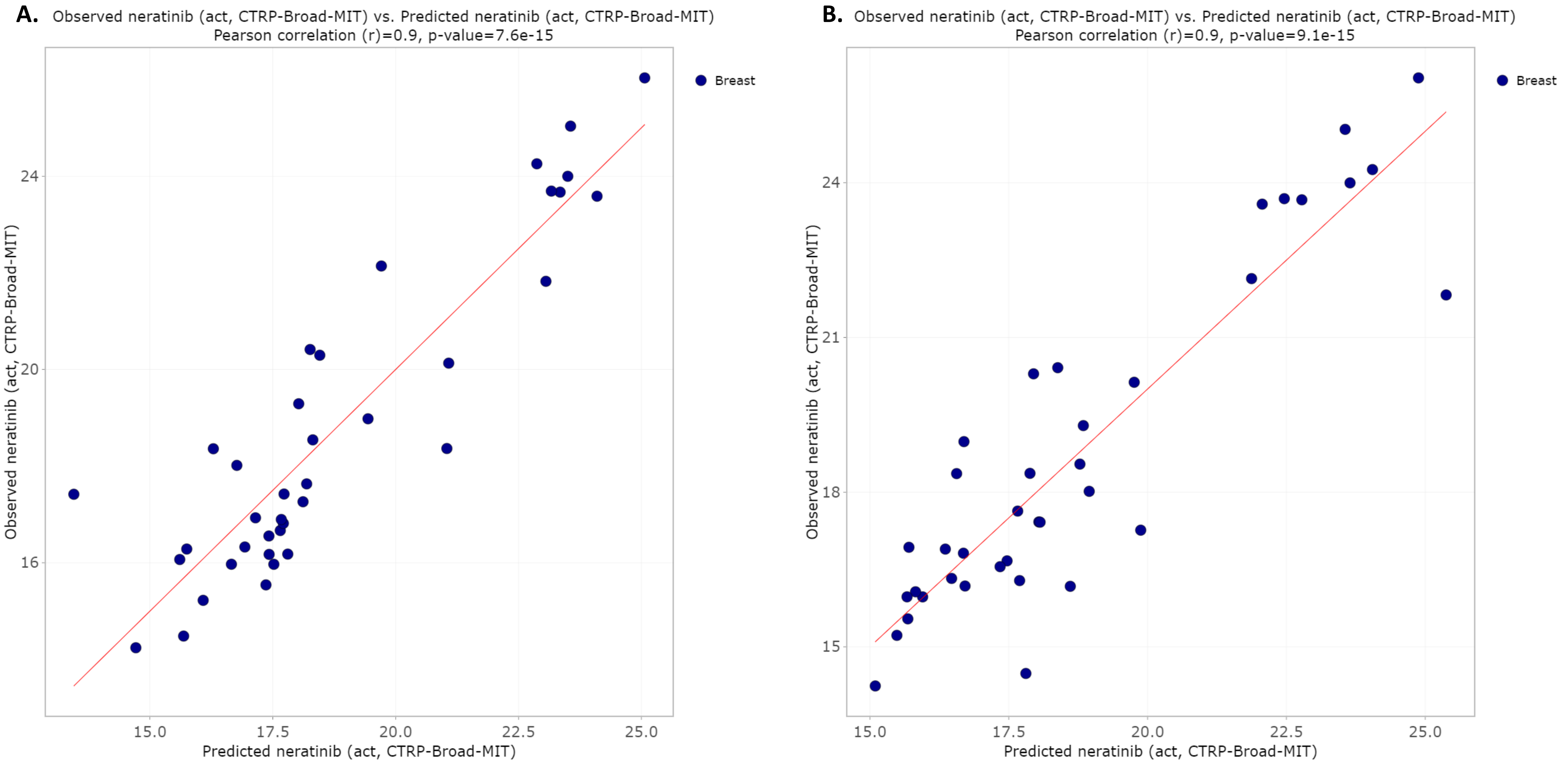

Figure S2: observed *versus* predicted response to neratinib in a pharmacogenomic model of 36 breast cancer cell lines, accessed *via* CellminerCDB. Correlations for (A) novel gene signature and (B) gene signature established by Desmedt *et al* 2008 (31) are shown.
